# Clinical characteristics and prognosis of patients with antiphospholipid antibodies based on cluster analysis: an 8-year cohort study

**DOI:** 10.1186/s13075-022-02814-w

**Published:** 2022-06-11

**Authors:** Wanting Qi, Jiuliang Zhao, Can Huang, Nan Jiang, Jing Li, Chanyuan Wu, Shangzhu Zhang, Chaojun Hu, Dong Xu, Qian Wang, Mengtao Li, Xinping Tian, Yan Zhao, Xiaofeng Zeng

**Affiliations:** 1 Department of Rheumatology and Clinical Immunology, Chinese Academy of Medical Sciences & Peking Union Medical College, Beijing, China; 2 National Clinical Research Center for Dermatologic and Immunologic Diseases (NCRC-DID), Ministry of Science & Technology, Beijing, China; 3 State Key Laboratory of Complex Severe and Rare Diseases, Peking Union Medical College Hospital (PUMCH), Beijing, China; 4 Key Laboratory of Rheumatology and Clinical Immunology, Ministry of Education, Beijing, China

**Keywords:** Antiphospholipid syndrome, Cluster analysis, Lupus erythematosus, Systemic, Heart disease risk factors, Morbidity

## Abstract

**Background:**

Antiphospholipid syndrome (APS) is an autoimmune disease characterized by persistent antiphospholipid antibodies (aPLs) positivity with a wide manifestation spectrum. A risk stratification is needed for management guidance and prognosis assessment. We aimed to identify phenotypes among aPL-positive patients and assess the prognosis of each phenotype.

**Methods:**

This was a single-center, prospective cohort study of aPL-positive patients presented to Peking Union Medical College Hospital from 2012 to 2020. Demographic characteristics, aPL-related manifestations, cardiovascular risk factors, and antibodies profiles were recorded. The primary endpoint was defined as a combination of newly onset thrombosis, major bleeding events, non-criteria manifestations, and all-cause death. Hierarchical cluster analysis and Kaplan-Meier survival analysis were performed.

**Results:**

Four clusters among 383 patients (70.2% female; mean age 37.7 years) were identified. Cluster 1 (*n* = 138): patients with systemic lupus erythematosus (SLE) and non-criteria manifestations; cluster 2 (*n* = 112): patients with multiple cardiovascular risk factors; cluster 3 (*n* = 83): female patients with obstetric morbidity; cluster 4 (*n* = 50): patients with isolated lupus anticoagulant (LA) positivity.

Non-criteria manifestations were found aggregated with SLE from cluster analysis of variables. Cluster 3 showed the best outcome, while cluster 2 suffered highest frenquency of newly onset arterial thrombosis.

**Conclusions:**

We identified 4 clinical phenotypes of aPL-positive patients. Non-criteria manifestations may indicate underlying SLE, for which immunosuppressive therapy besides anticoagulation may be necessary. Patients with isolated LA positivity suffered similar risks with secondary APS and patients with multiple cardiovascular risk factors. Attention should be paid to male patients, and the screening of cardiovascular risk factors should never be ignored.

**Supplementary Information:**

The online version contains supplementary material available at 10.1186/s13075-022-02814-w.

## Introduction

Antiphospholipid syndrome (APS) is an autoimmune disease characterized by persistent antiphospholipid antibodies (aPLs) positivity, leading to thrombotic events or obstetric morbidity. Despite considered as a rare disease with an annual incidence of 5 cases/100,000 and a low prevalence of 40~50 cases/100,000 [1], APS was responsible for 25–33% of early-onset (< 50 years old) cerebrovascular events, 15–30% of all deep venous thrombosis (DVT) episodes, and 10–15% of recurrent fetal loss [2]. It usually affected adults of reproductive age, with a female/male ratio of over 3:1 [3]. In addition to thrombotic events and recurrent obstetric losses, aPLs were also associated with a higher prevalence of thrombocytopenia, hemolytic anemia, heart valve disease, livedo reticularis, aPL-related nephropathy, and cognitive impairment, referred to “non-criteria manifestations,” which led to disease exacerbation [4, 5]. Therefore, the manifestation spectrum ranged from asymptomatic aPLs positivity, various non-criteria manifestations, obstetric morbidity, thrombosis, to life-threatening catastrophic APS (CAPS). The wide manifestation spectrum led to a heterogeneous entity and brought challenges to management of the syndrome.

As an exploratory method, cluster analysis was increasingly applied to APS [6–8]. Clusters corresponded to well-known phenotypes, including secondary APS, obstetric APS, asymptomatic aPLs carriers, and thrombotic APS with multiple cardiovascular risk factors, were identified [6–8]. However, the role of non-criteria manifestations in risk stratification was still poorly understood and there had been few reports on the prognosis of each phenotype. We aimed to develop a risk stratification based on cluster analysis integrating demographic characteristics, clinical manifestations, traditional cardiovascular risk factors, and antibodies profiles, to identify phenotypes among aPL-positive patients for management guidance and prognosis assessment.

## Methods

### Patients and data collection

This was a single-center, prospective cohort study conducted at Peking Union Medical College Hospital (PUMCH) from May 2012 to October 2020. The study included consecutive patients with persistent aPLs positivity (at least 12 weeks apart). Confirmed APS patients fulfilled 2006 Sydney APS Classification Criteria [9], while patients with a coexisting SLE fulfilled the 2019 European League Against Rheumatism (EULAR)/American College of Rheumatology Classification Criteria [10]. Confirmed APS patients were enrolled at the time of diagnosis, while event-free aPLs carriers were enrolled at the time of first aPLs positivity. Demographic characteristics, APS-related manifestations, traditional cardiovascular risk factors, and antibodies profiles were carefully collected at the baseline. Exclusive criteria were missing data. Study protocols were reviewed and approved by the Ethical Committee of PUMCH and informed consent was obtained from all patients.

### Antibody detection

Serum IgG/IgM anti-cardiolipin antibodies (aCL) and IgG/IgM anti-β2glycoprotein I antibodies (aβ2GPI) were detected by enzyme-linked immunosorbent assay (ELISA) (QUANTA Lite® ELISAs, INOVA Diagnostics, San Diego, CA, USA). The cutoff values for positivity were set as 40 IgG phospholipid (GPL) units or 40 IgM phospholipid (MPL) units. LA was detected by a traditional three-step procedure based on the guidelines of the International Society on Thrombosis and Hemostasis [9]. LA test positivity was defined as a prolonged diluted Russell viper venom time (dRVVT) in the screening step, which was not reversed by mixing with normal plasma but reversed by the addition of excess phospholipids in the confirmation step [11].

### Follow-up and outcomes

Patients were followed up every 3 to 6 months in outpatient clinics. Newly onset events (including non-criteria manifestations, thrombosis events, bleeding events, and death) and laboratory tests were collected. Updated follow-up information was obtained by contact with patients via telephone. The primary endpoint was determined as a combination of newly onset thrombotic events, non-criteria manifestations, major bleeding events, and all-cause deaths during follow-up. Newly onset thrombotic events were confirmed by computed tomographic angiography, magnetic resonance angiography, or digital subtraction angiography. Non-criteria manifestations included thrombocytopenia, hemolytic anemia, heart valve disease, aPL-related nephropathy, cognitive impairment, seizure, and chorea. Thrombocytopenia was defined as platelet count < 100 × 10^9^/L in the absence of other cause of thrombocytopenia. Heart valve disease was defined by the presence of valve thickness, valve vegetations, and/or valve dysfunction which met standards proposed by Amigo et al. [12]. APL-related nephropathy was biopsy-proven or clinically diagnosed based on manifestations such as hypertension, microscopic hematuria, proteinuria, and renal insufficiency in the absence of other secondary factors. Cognitive impairment, seizure, and chorea was diagnosed by professional neurologists based on clinical manifestations and head magnetic resonance imaging. Major bleeding events were defined as bleeding episodes which caused a hemoglobin decrease of ≥ 20 g/L within 24 h or an unplanned blood transfusion ≥ 2 U of red blood cells or whole blood. The observation period ended either at the primary endpoint or at the end of the study.

### Statistical analysis

Hierarchical cluster analysis with the Euclidean distance and the Ward method was applied to identify clusters of patients and variables separately. Characteristics used in the cluster analysis of patients included: SLE, male sex, smoking history, hypertension, body mass index (BMI) ≥ 25 kg/m^2^, arterial thrombosis (AT), deep venous thrombosis (DVT), early miscarriages, fetal death > 10th week, eclampsia, non-criteria manifestations, aCL, aβ2GPI, and LA. The “NbClust” package from R software provided 26 clustering criteria, and we decided the one with the most approval criteria as the optimal cluster number [13]. The Kruskal–Wallis test was applied among clusters for each variable. For multiple comparison, the Pearson chi-square test (or chi-square test with the Yates continuity, or Fisher exact test as appropriate) was used for categorical data, and the Mann-Whitney *U* test was used for qualitative data. Time to event curves were estimated by the Kaplan Meier method and compared using a two-side log-rank test. Alpha risk was set at 5% and the *P* value was adjusted according to Bonferroni correction. All statistical analysis was performed with R software (version 3.6.1; R Foundation for Statistical Computing, Vienna, Austria).

## Results

### Baseline characteristics

**Fig. 1:**
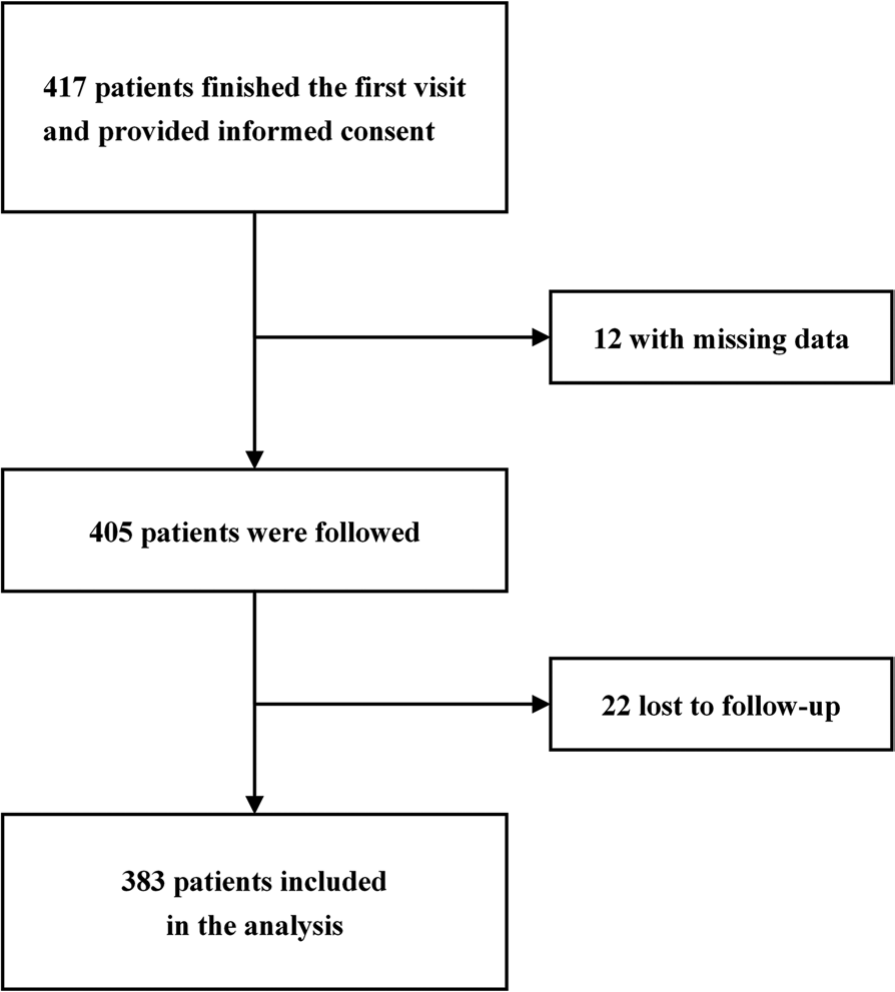
Flow diagram of the study

As shown in Fig. 1, 417 patients finished the first visit and provided informed consent. Twelve patients were excluded because of missing data. Twenty-two patients were lost to follow-up. A total of 383 patients (70.2% female; mean age 37.7 years) were included in the analysis, and the baseline characteristics were summarized in Table 1. They were followed for 3.0 ± 2.2 years, of whom 24.3% with a coexisting SLE. The mean age of onset was 31.3 years. Patients with a history of arterial thrombosis, deep venous thrombosis, and obstetric morbidity at baseline were 127 (33.2% of the total), 164 (42.8% of the total), and 142 (64.0% of female patients), respectively. Cluster analysis classified patients into 4 clusters (Supplementary Fig. 1). Multiple comparison of baseline characteristics among 4 clusters was shown in Table 1.

**Table 1:** Baseline characteristics and multiple comparison of aPL-positive patients in four clusters from cluster analysis

Variables, *n* (%)	All (*N* = 383)	Cluster 1 (*N* = 138)	Cluster 2 (*N* = 112)	Cluster 3 (*N* = 83)	Cluster 4 (*N* = 50)	*P*-value	*P*-value for multiple comparison					
							Cluster 1 vs Cluster 2	Cluster 1 vs Cluster 3	Cluster 1 vs Cluster 4	Cluster 2 vs Cluster 3	Cluster 2 vs Cluster 4	Cluster 3 vs Cluster 4
***Demographics***												
Male sex	114 (29.8)	8 (5.8)	87 (77.7)	1 (1.2)	18 (36.0)	0.000**	0.000*	0.186	0.000*	0.000*	0.000*	0.000*
Age (years), mean ± SD	37.7 ± 12.1	36.4 ± 11.4	39.7 ± 14.8	37.6 ± 9.8	36.9 ± 10.7	0.608	0.213	0.377	0.813	0.735	0.478	0.674
Age of onset ^a^ (years), mean ± SD	31.2 ± 12.0	29.1 ± 11.4	33.2 ± 14.6	32.7 ± 9.7	30.4 ± 10.0	0.008**	0.039	0.000*	0.223	0.471	0.536	0.179
Duration from onset to diagnosis (years), mean ± SD	2.5 ± 4.6	2.5 ± 4.7	2.8 ± 4.9	1.7 ± 2.8	3.4 ± 5.9	0.009**	0.011	0.566	0.052	0.008*	0.802	0.026
Asymptomatic aPLs carriers	45 (11.7)	21 (15.2)	9 (8.0)	9 (10.8)	6 (12.0)	0.368	0.082	0.358	0.578	0.503	0.61	0.838
Associated autoimmune diseases												
SLE	93 (24.3)	61 (44.2)	6 (5.4)	7 (8.4)	19 (38.0)	0.000**	0.000*	0.000*	0.000*	0.447	0.394	0.000*
Other autoimmune diseases^b^	12 (3.1)	3 (2.2)	3 (2.7)	3 (3.6)	3 (6.0)	0.591	0.591	1.000	0.833	0.396	1.000	0.559
***Clinical criteria***												
**Arterial thrombosis**	127 (33.2)	59 (42.8)	51 (45.5)	3 (3.6)	14 (28.0)	0.000**	0.659	0.000*	0.067	0.000*	0.035	0.000*
Cerebral infarction	70 (18.3)	38 (27.5)	26 (23.2)	3 (3.6)	3 (6.0)	0.000**	0.436	0.000*	0.002*	0.000*	0.008*	0.833
Coronary heart disease	18 (4.7)	8 (5.8)	9 (8.0)	0 (0.0)	1.000 (2.0)	0.045**	0.484	0.063	0.49	0.021	0.262	0.376
Lower limb arterial thrombosis	23 (6.0)	9 (6.5)	11 (9.8)	0 (0.0)	3 (6.0)	0.041**	0.339	0.043	1.000	0.009	0.619	0.098
Celiac artery thrombosis ^c^	20 (5.2)	3 (2.2)	12 (10.7)	1 (1.2)	4 (8.0)	0.005**	0.005*	0.998	0.153	0.008*	0.803	0.127
Retinal or ophthalmic artery thrombosis	9 (2.3)	6 (4.3)	2 (1.8)	0 (0.0)	1 (2.0)	0.205	0.433	0.134	0.753	0.509	1.000	0.376
**Venous thrombosis**	164 (42.8)	46 (33.3)	82 (73.2)	14 (16.9)	22 (44.0)	0.000**	0.000*	0.002*	0.769	0.000*	0.002*	0.003*
Lower limb deep venous thrombosis	110 (28.7)	33 (23.9)	58 (51.8)	6 (7.2)	13 (26.0)	0.000**	0.000*	0.009	0.401	0.000*	0.032	0.002*
Pulmonary embolism	82 (21.4)	23 (16.7)	44 (39.3)	4 (4.8)	11 (22.0)	0.000**	0.723	0.198	0.761	0.085	1.000	0.098
CTEPH	14 (3.7)	5 (3.6)	6 (5.4)	0 (0.0)	3 (6.0)	0.183	0.069	1.000	0.103	0.171	0.914	0.134
Celiac venous thrombosis ^d^	20 (5.2)	4 (2.9)	9 (8.0)	2 (2.4)	5 (10.0)	0.074	0.213	0.52	0.176	0.108	0.76	0.053
Intracranial venous sinus thrombosis	19 (5.0)	5 (3.6)	8 (7.1)	1 (1.2)	5 (10.0)	0.077	0.473	0.654	1.000	1.000	0.553	0.527
Retinal venous thrombosis	6 (1.6)	1 (0.7)	3 (2.7)	2 (2.4)	0 (0.0)	0.491	0.000*	0.002*	0.769	0.000*	0.002*	0.003*
**CAPS**	7 (1.8)	2 (1.4)	5 (4.5)	0 (0.0)	0 (0.0)	0.118	0.293	0.529	1.000	0.136	0.305	1.000
**Obstetric morbidity** ^e^	142 (64.0)	60 (60.0)	4 (23.5)	63 (81.8)	15 (53.6)	0.000**	0.005*	0.002*	0.542	0.000*	0.048	0.003*
Early miscarriages	77 (34.5)	36 (36.0)	1 (5.9)	34 (43.6)	6 (21.4)	0.011**	0.014	0.304	0.147	0.003*	0.332	0.038
Fetal death > 10th week	64 (28.7)	24 (24.0)	1 (5.9)	29 (37.2)	10 (35.7)	0.032**	0.172	0.056	0.215	0.012	0.057	0.89
Premature birth ^f^	34 (15.2)	19 (19.0)	4 (23.5)	8 (10.3)	3 (10.7)	0.268	0.917	0.107	0.457	0.276	0.468	1
**Non-criteria manifestations**	184 (48.0)	95 (68.8)	54 (48.2)	12 (14.5)	23 (46.0)	0.000**	0.001*	0.000*	0.004*	0.000*	0.794	0.000*
Thrombocytopenia	152 (39.7)	80 (58.0)	40 (35.7)	11 (13.3)	21 (42.0)	0.000**	0.000*	0.000*	0.052	0.000*	0.446	0.000*
Hemolytic anemia	48 (12.5)	29 (21.0)	10 (8.9)	2 (2.4)	7 (14.0)	0.000**	0.009	0.000*	0.28	0.061	0.331	0.026
Heart valve disease	22 (5.7)	13 (9.4)	5 (4.5)	2 (2.4)	2 (4.0)	0.008**	0.132	0.045	0.364	0.709	1.000	1.000
APL-associated nephropathy	17 (4.4)	5 (3.6)	11 (9.8)	0 (0.0)	1 (2.0)	0.006**	0.046	0.198	0.928	0.009	0.152	0.376
Livedo reticularis	7 (1.8)	7 (5.1)	0 (0.0)	0 (0.0)	0 (0.0)	0.005**	0.042	0.091	0.235	1.000	1.000	1.000
Skin ulcer	2 (0.5)	1 (0.7)	1 (0.9)	0 (0.0)	0 (0.0)	1.000	1.000	1.000	1.000	1.000	1.000	1.000
Non-stroke CNS manifestation	24 (6.3)	15 (10.9)	7 (6.2)	1 (1.2)	1 (2.0)	0.017**	0.2	0.007*	0.103	0.164	0.447	1.000
Cognitive impairment	10 (2.6)	7 (5.1)	3 (2.7)	0 (0.0)	0 (0.0)	0.077	0.525	0.091	0.235	0.361	0.553	1.000
Seizure	17 (4.4)	10 (7.2)	5 (4.5)	1 (1.2)	1 (2.0)	0.15	0.357	0.093	0.316	0.377	0.751	1.000
Chorea	5 (1.3)	4 (2.9)	1 (0.9)	0 (0.0)	0 (0.0)	0.394	0.501	0.296	0.519	1.000	1.000	1.000
***Cardiovascular risk factors***												
Body mass index (kg/m^2^), mean ± SD	23.7 ± 4.0	23.6 ± 4.3	24.7 ± 3.7	22.6 ± 3.9	23.6 ± 3.4	0.000**	0.006*	0.128	0.831	0.000*	0.056	0.151
Smoking (past or current)	69 (18.0)	3 (2.2)	51 (45.5)	4 (4.8)	11 (22.0)	0.000**	0.000*	0.49	0.000*	0.000*	0.004*	0.002*
Hypertension	63 (16.4)	18 (13.0)	40 (35.7)	1 (1.2)	4 (8.0)	0.000**	0.000*	0.002*	0.342	0.000*	0.000*	0.127
Coronary artery disease	14 (3.7)	5 (3.6)	9 (8.0)	0 (0.0)	0 (0.0)	0.011**	0.131	0.198	0.395	0.021	0.091	1.000
Diabetes	10 (2.6)	6 (4.3)	4 (3.6)	0 (0.0)	0 (0.0)	0.132	1.000	0.134	0.303	0.219	0.421	1.000
Dyslipidemia	211 (55.1)	73 (52.9)	65 (58.0)	44 (53.0)	29 (58.0)	0.807	0.417	0.987	0.535	0.485	0.997	0.576
CHO > 5.70 mmol/L	85 (22.2)	20 (14.5)	23 (20.5)	30 (36.1)	12 (24.0)	0.002**	0.208	0.000*	0.125	0.015	0.621	0.144
TG > 1.70 mmol/L	182 (47.5)	66 (47.8)	50 (44.6)	39 (47.0)	27 (54.0)	0.746	0.616	0.904	0.454	0.745	0.271	0.433
LDL > 3.37 mmol/L	83 (21.7)	16 (11.6)	31 (27.7)	22 (26.5)	14 (28.0)	0.005**	0.001*	0.004*	0.007*	0.856	0.966	0.851
Hcy > 15 μmol/L	89 (23.2)	22 (15.9)	47 (42.0)	7 (8.4)	13 (26.0)	0.000**	0.000*	0.109	0.117	0.000*	0.052	0.006*
***Antibody profile***												
aCL, IgG/IgM	186 (48.6)	85 (61.6)	67 (59.8)	31 (37.3)	3 (6.0)	0.000**	0.775	0.000*	0.000*	0.002*	0.000*	0.000*
aβ2-GPI, IgG/IgM	266 (69.5)	109 (79.0)	88 (78.6)	68 (81.9)	1 (2.0)	0.000**	0.937	0.596	0.000*	0.562	0.000*	0.000*
LA	283 (73.9)	131 (94.9)	99 (88.4)	4 (4.8)	49 (98.0)	0.000**	0.058	0.000*	0.608	0.000*	0.088	0.000*
ANA	151 (39.4)	82 (59.4)	29 (25.9)	17 (20.5)	23 (46.0)	0.000*	0.000*	0.000*	0.102	0.379	0.011	0.002*
Anti-dsDNA	65 (17.0)	39 (28.3)	8 (7.1)	5 (6.0)	13 (26.0)	0.000**	0.000*	0.000*	0.759	0.757	0.001*	0.001*
aCL, IgG/IgM + aβ2-GPI, IgG/IgM	160 (41.8)	80 (58.0)	57 (50.9)	23 (27.7)	0 (0.0)	0.000**	0.263	0.000*	0.000*	0.001*	0.000*	0.000*
LA+ aCL, IgG/IgM	148 (38.6)	83 (60.1)	60 (53.6)	2 (2.4)	3 (6.0)	0.000**	0.296	0.000*	0.000*	0.000*	0.000*	0.559
LA+ aβ2-GPI, IgG/IgM	187 (48.8)	104 (75.4)	78 (69.6)	4 (4.8)	1 (2.0)	0.000**	0.312	0.000*	0.000*	0.000*	0.000*	0.721
Multiple aPL positive	229 (59.8)	109 (79.0)	91 (81.2)	25 (30.1)	4 (8.0)	0.000**	0.656	0.000*	0.000*	0.000*	0.000*	0.003*
Triple aPL positive	133 (34.7)	79 (57.2)	52 (46.4)	2 (2.4)	0 (0.0)	0.000**	0.089	0.000*	0.000*	0.000*	0.000*	0.527
***Other laboratory tests***												
ESR elevation ^g^	106 (27.7)	45 (32.6)	29 (25.9)	15 (18.1)	17 (34.0)	0.083	0.247	0.019	0.858	0.196	0.29	0.037
CRP > 8 mg/L	53 (13.8)	22 (15.9)	18 (16.1)	4 (4.8)	9 (18.0)	0.061	0.978	0.013	0.737	0.014	0.761	0.029
C3 < 0.730 g/L	60 (15.7)	32 (23.2)	12 (10.7)	10 (12.0)	6 (12.0)	0.025**	0.01	0.041	0.091	0.771	0.81	0.993
C4 < 0.100 g/L	60 (15.7)	33 (23.9)	12 (10.7)	8 (9.6)	7 (14.0)	0.009**	0.007*	0.008*	0.142	0.807	0.548	0.441

**P < 0.05: Kruskal–Wallis test

*P < 0.0083: Pearson chi-square test (or chi-square test with the Yates continuity, or Fisher exact test if appropriate) for categorical data, and the Mann-Whitney *U* test for qualitative data after *Bonferroni* correction

*SD* standard deviation, *CTEPH* chronic thromboembolic pulmonary hypertension, *CNS* central nervous system, *CHO* total cholesterol, *TG* triglycerides, *LDL* low-density lipoprotein, *Hcy* homocysteine, *ESR* erythrocyte sedimentation rate, *CRP* C-reactive protein

^a^Age of onset was defined as the age at first thrombotic or obstetric episode for confirmed APS patients, or at the first aPLs positivity for asymptomatic aPLs carriers

^b^Other autoimmune disease included primary Sjogren’s syndrome, systemic vasculitis, and psoriasis

^c^Celiac artery thrombosis included thrombosis involving abdominal aorta, mesenteric artery, splenic artery, renal artery, and adrenal artery

^d^Celiac venous thrombosis included thrombosis involving portal vein, hepatic vein, splenic vein, mesenteric vein, inferior vena cava, and renal vein

^e^Calculated in female patients

^f^Premature birth due to preeclampsia, eclampsia, or placental insufficiency before 34th week of gestation

^g^ESR elevation was defined as ESR > 15 mm/h in males or > 20 mm/h in females

### Cluster 1

Cluster 1 included 138 patients (36.0% of the total), 44.2% with a coexisting SLE. Non-criteria manifestations, especially thrombocytopenia, hemolytic anemia, heart valve disease, livedo reticularis, and non-stroke center nervous system (CNS) manifestations (including cognitive impairment, seizure and chorea), presented the most in cluster 1. Cluster 1 presented with high AT rate (42.8%) and moderate DVT (33.3%) rate, with 57.2% positive of triple aPLs.

### Cluster 2

Cluster 2 (112 patients, 29.2% of the total) represented male patients with multiple cardiovascular risk factors, of whom 77.7% were male, 45.5% with a smoking history, 35.7% with hypertension, 42.0% with hyperhomocysteinemia and the mean BMI was 24.7 kg/m^2^. Cluster 2 showed the highest rate of AT (45.5%) and DVT (73.2%), and moderate rate of non-criteria manifestations (48.2%), with 46.4% positive of triple aPLs.

### Cluster 3

Women with obstetric morbidity were aggregated in cluster 3 (83 patients, 21.7% of the total), in which 43.6% with history of early miscarriages, 37.2% with fetal death > 10th week, 10.3% with premature birth of fetus. Twenty-five patients (30.1%) were positive for more than one aPL, while only 2 patients (2.4%) were triple aPLs positive. The proportions of AT (3.6%), DVT (16.9%), and non-criteria manifestations (14.5%) were all the lowest in cluster 3.

### Cluster 4

Cluster 4 represented patients with isolated LA positivity (98.0%). Fifty patients (13.1%) were included, with 36.0% being male and 38.0% coexisting with SLE. High AT rate (28.0%) and moderate DVT (44.0%) and non-criteria manifestations rates (46.0%) were shown in cluster 4.

### Cluster analysis of variables

**Fig. 2:**
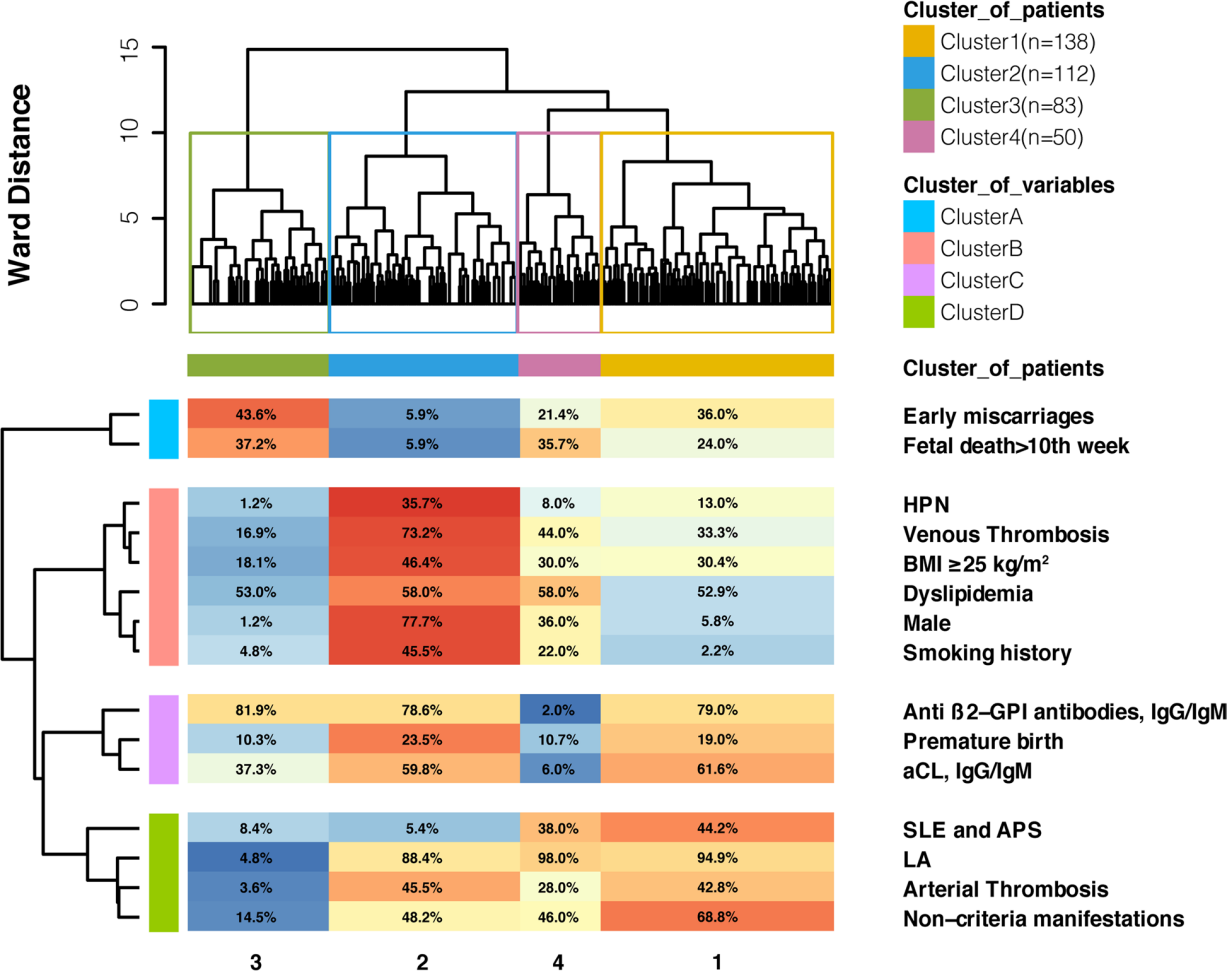
Hierarchical cluster analysis of 383 aPL-positive patients (*x*-axis) and 15 variables (*y*-axis) with the Euclidean distance and the Ward method. Four clusters of patients (cluster 1, 2, 3, 4) and four clusters of variables (cluster A, B, C, D) were identified separately. HPN, hypertension; BMI, body mass index; SLE, systemic lupus erythematosus; aCL, anticardiolipin antibodies; LA, lupus anticoagulant

Four clusters of variables were identified (Fig. 2): (A) early miscarriages and fetal death > 10th week; (B) venous thrombosis, male sex, smoking history, hypertension, dyslipidemia and BMI ≥ 25 kg/m^2^; (C) premature birth, aCL and aβ2-GPI; and (D) arterial thrombosis, LA, SLE and non-criteria manifestations.

### Follow-up

**Fig. 3:**
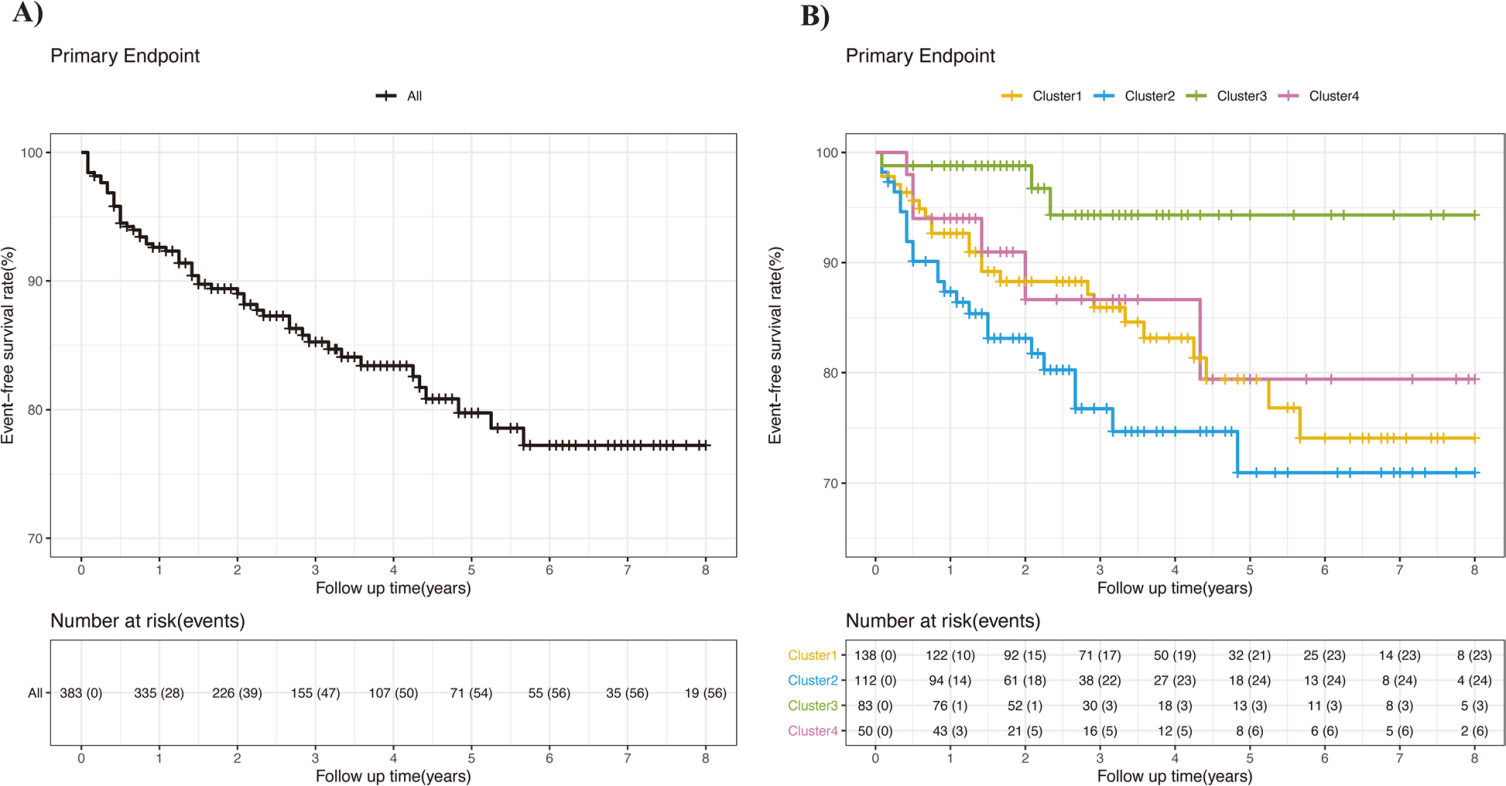
**A** Cumulative event-free survival curves in 383 aPL-positive patients. **B** Cumulative event-free survival curves of four clusters. Cluster 1: secondary APS; cluster 2: male patients with multiple cardiovascular risk factors; cluster 3: obstetric morbidity; cluster 4: isolated LA positivity

**Fig. 4:**
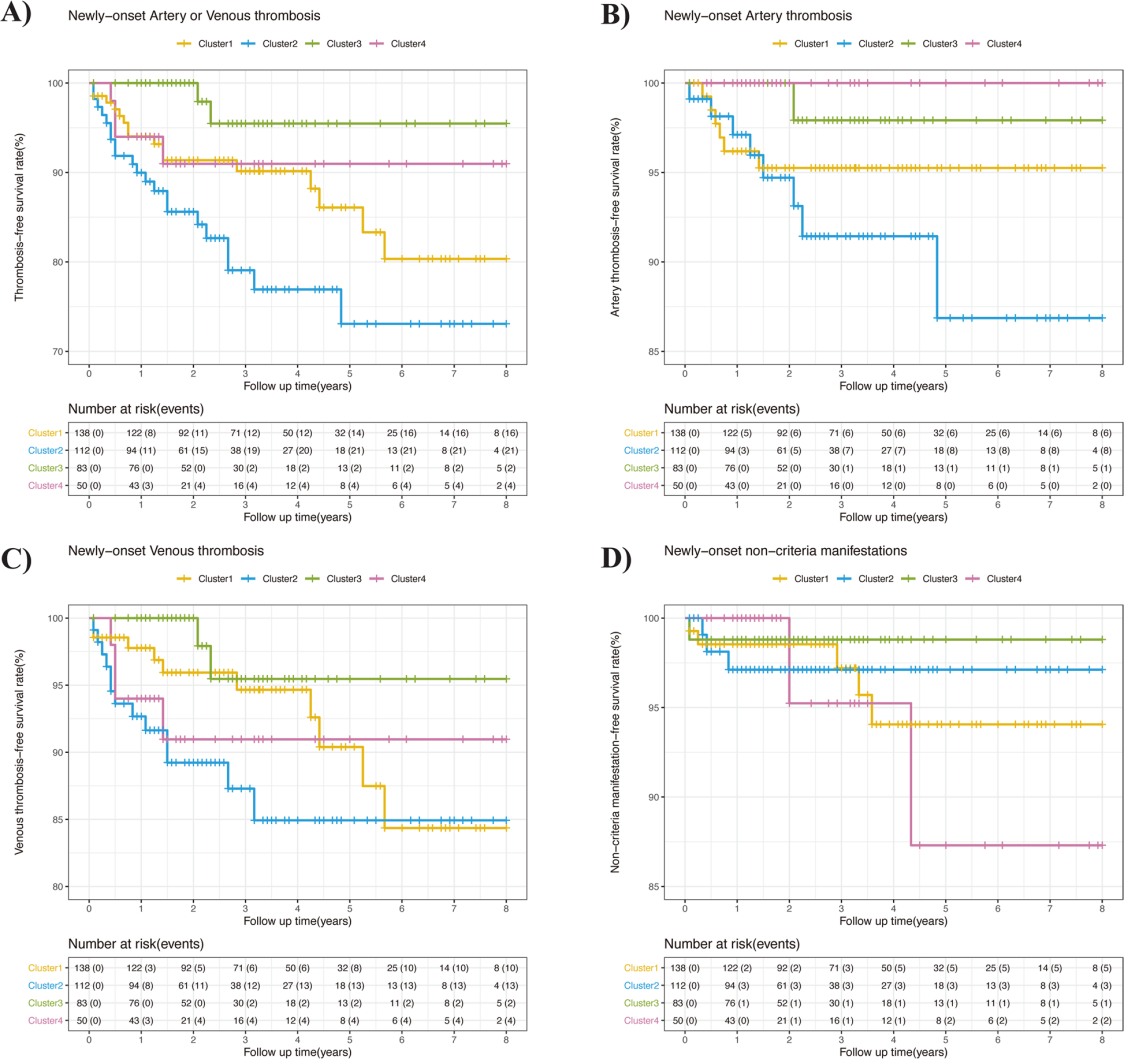
**A** Cumulative thrombosis-free survival curves of four clusters. **B** Cumulative AT-free survival curves of four clusters. **C** Cumulative DVT-free survival curves of four clusters. **D** Cumulative non-criteria manifestation-free survival curves of four clusters. AT, arterial thrombosis; DVT, deep venous thrombosis. Cluster 1: secondary APS; cluster 2: male patients with multiple cardiovascular risk factors; cluster 3: obstetric morbidity; cluster 4: isolated LA positivity

The mean follow-up was 36.4 months. Primary endpoint occurred in 56 patients, with an event occurrence rate of 4.82 per 100 person-years (Supplementary Table 1). From Kaplan Meier analysis, 1-, 3-, and 5-year event-free survival rates were 92.6% (95% confidence interval [CI], 90–95.3%), 85.2% (95% CI, 81.3–89.4%) and 79.8% (95% CI, 74.4–85.5%), respectively (Fig. 3, Supplementary Table 2). Clusters 1, 2, 3, and 4 showed the 5-year event-free survival rate of 79.4% (95% CI, 71.3–88.4%), 71.0% (95% CI, 60.3–83.5%), 94.3% (95% CI, 88.1–100%), and 79.4% (95% CI, 63.9–98.7%), respectively (Fig. 3, Supplementary Table 2). For primary endpoint and thrombosis endpoint, patients in cluster 3 showed the lowest risks, while patients in clusters 1, 2, and 4 suffered similar risks (Fig. 4, Supplementary Table 2). For the AT endpoint, cluster 2 showed significant higher rate (2.57 per 100 person-years) than the other clusters (Fig. 4, Supplementary Table 2). For endpoints of DVT, non-criteria manifestations, major bleeding events, or mortality, no difference was found among clusters.

## Discussion

This single-center prospective cohort study with 383 aPL-positive patients identified 4 clusters with different combination of clinical features, which reflected the heterogeneity of the syndrome. Cluster 1: secondary APS (SAPS) with non-criteria manifestations; cluster 2: male patients with multiple cardiovascular risk factors; cluster 3: female patients with obstetric morbidity; cluster 4: patients with isolated LA positivity. Another four clusters were identified from cluster analysis of variables, and non-criteria manifestations were found aggregated with SLE in both cluster analysis. Patients with isolated LA positivity suffered similar risk of primary endpoint with SAPS and patients with multiple cardiovascular risk factors.

Cluster 1 represented SAPS and aggregated with non-criteria manifestations, especially thrombocytopenia, hemolytic anemia, heart valve disease, livedo reticularis, and non-stroke CNS manifestations. From cluster analysis of variables, non-criteria manifestations were once again found together with SLE. Similar results were reported in cluster analysis of an international cohort, in which aPL-related nephropathy, thrombocytopenia, and hemolytic anemia were found aggregated with secondary APS [8]. In previous studies, an increased incidence of thrombocytopenia, hemolytic anemia, heart valve disease, livedo reticularis, skin ulcers, pseudovascolitis, and chorea was observed in aPL-positive patients with SLE compared with those without SLE [4, 14–17]. We considered these non-criteria manifestations more supportive of thrombotic microangiopathy in target organs already compromised by inflammatory damage of SLE. For heart valve disease, the immune complex involving aCL, aβ2GPI, and complement was deposited on the subendothelial heart valve, and on this basis, aPLs promoted thrombosis and further valve damage [12]. From the clinical perspective, the heart valve disease was progressive despite anticoagulation [18]. Clinicians should be alert to the underlying SLE in patients with those non-criteria manifestations, for whom anticoagulants alone may offer insufficient protection [19] and for those with a severe condition immunosuppressive therapy besides anticoagulation may be necessary. Further search is needed to investigate whether non-criteria manifestations can predict future SLE in aPL-positive patients, while it is certain that non-criteria manifestations should be taken into account in the APS assessment [20].

Cluster 2 in our study represented patients with multiple well-known cardiovascular risk factors, as another major concern in primary APS for clinicians. The 5-year event-free survival rates in clusters 2 were 71.0%, similar to that of 74.9% reported in the Japanese cohort [6]. It showed the highest rate of both arterial and venous thrombosis at baseline and the highest incidence of primary endpoint and newly onset thrombosis during follow-up. From cluster analysis of variables, venous thrombosis was aggregated with male sex, smoking history, hypertension, dyslipidemia, and BMI ≥ 25 kg/m^2^, which were all well-proven venous thrombosis and atherosclerosis risk factors [21–23]. For atherosclerosis, increasing evidence suggested that its pathophysiology involved autoimmune mechanisms [24, 25]. Accelerated atherosclerosis and thrombosis associated with aPLs may directly lead to acute cardiovascular events. Males were not predisposed to APS, but to atherosclerosis [23]. Male APS patients tended to have more cardiovascular risk factors and suffered a higher risk of arterial thrombosis. For APS patients, especially for males or for those with a high-risk antibody profile, anticoagulation should be performed under adequate management of current, modifiable cardiovascular risk factors.

In addition to clusters corresponded to well-known subtypes, we identified a cluster (cluster 4) characterized by isolated LA positivity. To the best our knowledge, this is the first time that patients with isolated LA positivity were identified in a distinct cluster, facilitating the risk assessment of LA. In the early 1950s, lupus anticoagulant was first coined to described the “peculiar hemorrhagic disorder” found in SLE patients [26]. Interestingly, LA was subsequently found associated with thrombosis rather than bleeding, since LA were actually immunoglobulins targeting phospholipid binding protein on cell membranes, prothrombin, and β2GPI [27, 28]. LA positivity was defined as one of the high-risk aPLs profiles according to the EULAR recommendations [22] and was assigned of 4 points in the Global Anti-Phospholipid Syndrome Score (GAPSS) [15]. In 2014, Reynaud et al. [29] published a meta-analysis with 16,441 patients from 30 studies to quantify the thrombotic risk associated with each aPL. They reported odds ratio of 6.14 (95% confidence interval CI 2.74–13.8, *P* < 0.001) for venous thrombosis associated with LA, compared with odds ratio of 1.46 and 1.61 for aCL and aβ2GPI, respectively. For arterial thrombosis, the odds ratio of LA was 3.58 (95% CI 1.29–9.92, *P* = 0.01). LA was identified as an independent risk factor of first thrombosis episode in aPLs carriers [30]. In our study, cluster 4 showed the shortest Ward distance with cluster 1, indicating the lowest inter-group differences with SAPS. From multiple comparison, as compared with cluster 1, cluster 4 aggregated more males with smoking history and high LDL levels and less patients with history of stroke or non-criteria manifestations. LA was aggregated with arterial thrombosis in cluster D. From Kaplan-Meier survival analysis, cluster 4 shared similar prognosis with cluster 1 and cluster 2 in terms of primary endpoint, confirming that LA represented a high-risk antibody spectrum. LA-positive patients may suffer similar risks with SAPS and patients with multiple cardiovascular risk factors.

Our study has some limitations. Firstly, this was a single-center study conducted in a tertiary hospital. The enrichment of difficult cases may introduce selection bias and further multi-center studies were needed to confirm the results. Patients with SLE were included in the study, who were prone to have some non-criteria manifestations, such as thrombocytopenia and hemolytic anemia, which might led to a bias and limited the extrapolability and informative value of cluster 1. As an exploratory tool, cluster analysis was not able to identify dependent and independent risk factors for the primary endpoint, but was a suitable methodology for this entity with great heterogeneity. Further quantitative analysis could be conducted in each cluster. The treatment was not included in the variables due to the large individual differences in therapies, especially for patients with SLE and pregnant women. The absence of therapies limited prognostic values of clusters. A treatment-naïve inception cohort is needed for further assessment of the prognostic difference.

## Conclusions

In conclusion, we identified 4 clinical phenotypes of aPL-positive patients derived from hierarchical cluster analysis. The comparison among these clusters revealed the heterogeneity of APS. APS secondary to SLE was always aggregated with non-criteria manifestations. Therefore, clinicians should be alert to the possibility of SLE in aPL-positive patients with coexisting non-criteria manifestations, for whom immunosuppressive therapy besides anticoagulation may be necessary. Cluster 4 represented patients with isolated LA positivity and shared similar prognosis with secondary APS and male patients with multiple cardiovascular risk factors, which confirmed that LA represented a high-risk antibody spectrum. Additionally, cardiovascular risk factors played an important role in both arterial and venous thrombosis events and led to poor prognosis. Therefore, more attention should be paid to male patients, and the screening and management of cardiovascular risk factors should not be ignored.

## Supplementary Information

**Additional file 1: Supplementary Figure 1.** Number of clusters approved by 26 clustering criteria from “NbClust” package of R software. **Supplementary Table 1.** Events and event-free Kaplan-Meier survival rate of aPL-positive patients in four clusters from cluster analysis. **Supplementary Table 2.** Event-free survival rate and 95% confidence interval from Kaplan-Meier survival analysis.

## Data Availability

The dataset used and analyzed during the current study was available from the corresponding author on reasonable request.

## Abbreviations

APS: Antiphospholipid syndrome
aPLs: Antiphospholipid antibodies
SLE: Systemic lupus erythematosus
LA: Lupus anticoagulant
DVT: Deep venous thrombosis
CAPS: Catastrophic antiphospholipid syndrome
PUMCH: Peking Union Medical College Hospital
EULAR: European League Against Rheumatism
aCL: Anti-cardiolipin antibodies
aβ2GPI: Anti-β2glycoprotein I antibodies
ELISA: Enzyme-linked immunosorbent assay
GPL: IgG phospholipid
MPL: IgM phospholipid
dRVVT: Diluted Russell viper venom time
BMI: Body mass index
AT: Arterial thrombosis
CNS: Central nervous system
CI: Confidence interval
SAPS: Secondary antiphospholipid syndrome
GAPSS: Global Anti-Phospholipid Syndrome Score
