# Supplementary material for: Clinical characteristics and prognosis of patients with antiphospholipid antibodies based on cluster analysis: an 8-year cohort study
Source: Arthritis Res Ther. 2022 Jun 11;24:140. doi: 10.1186/s13075-022-02814-w (PMC9188169; doi:10.1186/s13075-022-02814-w)
Supplement: Supplementary file 1 — Additional file 1: Supplementary Figure 1. Number of clusters approved by 26 clustering criteria from “NbClust” package of R software. Supplementary Table 1. Events and event-free Kaplan-Meier survival rate of aPL-positive patients in four clusters from cluster analysis. Supplementary Table 2. Event-free survival rate and 95% confidence interval from Kaplan-Meier survival analysis. [file 13075_2022_2814_MOESM1_ESM.docx]

Supplementary Figure 1. Number of clusters approved by 26 clustering criteria from “NbClust” package of R software. The optimal cluster number with the most approval criteria was four.

Supplementary Table 1. Events and event-free Kaplan-Meier survival rate of aPL-positive patients in four clusters from cluster analysis.

| Variables, n (%) | All  (N=383) | Cluster 1  (N=138) | Cluster 2  (N=112) | Cluster 3  (N=83) | Cluster 4  (N=50) | *P*-Value |
| --- | --- | --- | --- | --- | --- | --- |
| Observation time (year), mean ± SD | 3.0 ± 2.2 | 3.4 ± 2.3 | 2.8 ± 2.1 | 3.0 ± 2.1 | 2.7 ± 2.2 | 0.047* |
| Antiplatelet agents | 247 (64.5) | 94 (68.1) | 51 (45.5) | 71 (85.5) | 31 (62.0) | 0.000* |
| Anticoagulant | 276 (72.1) | 98 (71.0) | 92 (82.1) | 50 (60.2) | 36 (72.0) | 0.009* |
| Glucocorticoid | 202 (52.7) | 93 (67.4) | 53 (47.3) | 27 (32.5) | 29 (58.0) | 0.000* |
| Hydroxychloroquine | 325 (84.9) | 124 (89.9) | 87 (77.7) | 73 (88.0) | 41 (82.0) | 0.044* |
| Other immunosuppressive agents | 179 (46.7) | 92 (66.7) | 57 (50.9) | 7 (8.4) | 23 (46.0) | 0.000* |
| Primary endpoint | 56 (14.6) | 23 (16.7) | 24 (21.4) | 3 (3.6) | 6 (12.0) | 0.005* |
| Thrombosis | 43 (11.2) | 16 (11.6) | 21 (18.8) | 2 (2.4) | 4 (8.0) | 0.004* |
| Arterial thrombosis | 15 (3.9) | 6 (4.3) | 8 (7.1) | 1 (1.2) | 0 (0.0) | 0.078 |
| Cerebral infarction | 10 (2.6) | 4 (2.9) | 5 (4.5) | 1 (1.2) | 0 (0.0) | 0.315 |
| Coronary heart disease | 2 (0.5) | 0 (0.0) | 2 (1.8) | 0 (0.0) | 0 (0.0) | 0.282 |
| Carotid artery thrombosis | 1 (0.3) | 1 (0.7) | 0 (0.0) | 0 (0.0) | 0 (0.0) | 1 |
| Celiac artery thrombosis ^a^ | 2 (0.5) | 1 (0.7) | 0 (0.0) | 1 (1.2) | 0 (0.0) | 0.789 |
| Lower limb arterial thrombosis | 1 (0.3) | 0 (0.0) | 1 (0.9) | 0 (0.0) | 0 (0.0) | 0.64 |
| Retinal or ophthalmic artery thrombosis | 1 (0.3) | 0 (0.0) | 1 (0.9) | 0 (0.0) | 0 (0.0) | 0.64 |
| Venous thrombosis | 29 (7.6) | 10 (7.2) | 13 (11.6) | 2 (2.4) | 4 (8.0) | 0.122 |
| Pulmonary embolism | 19 (5.0) | 7 (5.1) | 10 (8.9) | 0 (0.0) | 2 (4.0) | 0.043* |
| Lower limb deep venous thrombosis | 8 (2.1) | 2 (1.4) | 2 (1.8) | 1 (1.2) | 3 (6.0) | 0.223 |
| Upper limb deep venous thrombosis | 1 (0.3) | 1 (0.7) | 0 (0.0) | 0 (0.0) | 0 (0.0) | 1 |
| Celiac venous thrombosis ^b^ | 2 (0.5) | 1 (0.7) | 0 (0.0) | 1 (1.2) | 0 (0.0) | 0.789 |
| Intracranial venous sinus thrombosis | 1 (0.3) | 0 (0.0) | 1 (0.9) | 0 (0.0) | 0 (0.0) | 0.64 |
| Retinal venous thrombosis | 1 (0.3) | 0 (0.0) | 1 (0.9) | 0 (0.0) | 0 (0.0) | 0.64 |
| Non-criteria manifestations | 11 (2.9) | 5 (3.6) | 3 (2.7) | 1 (1.2) | 2 (4.0) | 0.717 |
| Thrombocytopenia | 2 (0.5) | 2 (1.4) | 0 (0.0) | 0 (0.0) | 0 (0.0) | 0.632 |
| Hemolytic anemia | 3 (0.8) | 1 (0.7) | 1 (0.9) | 0 (0.0) | 1 (2.0) | 0.571 |
| Heart valve disease | 1 (0.3) | 0 (0.0) | 0 (0.0) | 1 (1.2) | 0 (0.0) | 0.347 |
| APS-related nephropathy | 1 (0.3) | 0 (0.0) | 0 (0.0) | 0 (0.0) | 1 (2.0) | 0.131 |
| Non-stroke CNS manifestations | 2 (0.5) | 2 (1.4) | 0 (0.0) | 0 (0.0) | 0 (0.0) | 0.632 |
| Diffuse alveolar hemorrhage | 1 (0.3) | 0 (0.0) | 1 (0.9) | 0 (0.0) | 0 (0.0) | 0.64 |
| Coronary artery microthrombosis | 1 (0.3) | 0 (0.0) | 1 (0.9) | 0 (0.0) | 0 (0.0) | 0.64 |
| Major bleeding events | 1 (0.3) | 0 (0.0) | 1 (0.9) | 0 (0.0) | 0 (0.0) | 0.64 |
| Death | 4 (1.0) | 2 (1.4) | 1 (0.9) | 1 (1.2) | 0 (0.0) | 1 |
| Primary endpoint occurrence rate per 100 person-years | 4.82 | 4.90 | 7.72 | 1.21 | 4.50 | 0.013* |
| Thrombosis rate per 100 person-years | 3.70 | 3.41 | 6.75 | 0.81 | 3.00 | 0.002* |
| AT rate per 100 person-years | 1.29 | 1.28 | 2.57 | 0.40 | 0 | 0.039* |
| DVT rate per 100 person-years | 2.50 | 2.13 | 4.18 | 0.81 | 3.00 | 0.100 |
| Newly-onset non-criteria manifestations rate per 100 person-years | 0.95 | 1.07 | 0.96 | 0.40 | 1.50 | 0.512 |
| Major bleeding rate per 100 person-years | 0.09 | 0 | 0.32 | 0 | 0 | 0.416 |
| Mortality rate per 100 person-years | 0.34 | 0.43 | 0.32 | 0.4 | 0 | 0.751 |

**P*＜0.05: Kruskal–Wallis test.

*SD*, standard deviation; *AT*, arterial thrombosis; *DVT*, deep venous thrombosis; *CI*, confidence interval.

^a^ Celiac artery thrombosis included thrombosis involving abdominal aorta and mesenteric artery.

^b^ Celiac venous thrombosis included thrombosis involving portal vein and hepatic vein.

Supplementary Table 2. Event-free survival rate and 95% confidence interval from Kaplan-Meier survival analysis.

| Event-free survival rate, mean (95% CI) | | All  (N=383) | Cluster 1  (N=138) | Cluster 2  (N=112) | Cluster 3  (N=83) | Cluster 4  (N=50) | *P*-Value |
| --- | --- | --- | --- | --- | --- | --- | --- |
| Primary endpoint | 1-year | 92.6%  （90%-95.3%) | 92.6%  （88.4%-97.1%) | 87.4%  （81.4%-93.8%) | 98.8%  （96.5%-100%) | 94%  （87.6%-100%) | 0.013* |
|  | 3-year | 85.2%  (81.3%-89.4%) | 85.9%  (79.8%-92.5%) | 76.8%  (68.3%-86.2%) | 94.3%  (88.1%-100%) | 86.6%  (75.7%-99.1%) |  |
|  | 5-year | 79.8%  （74.4%-85.5%) | 79.4%  （71.3%-88.4%) | 71%  （60.3%-83.5%) | 94.3%  （88.1%-100%) | 79.4%  （63.9%-98.7%) |  |
| Thrombosis endpoint | 1-year | 94.1%  （91.8%-96.5%) | 94%  （90.1%-98.1%) | 90%  （84.5%-95.8%) | 100%  （100%-100%) | 94%  （87.6%-100%) | 0.002* |
|  | 3-year | 88.2%  (84.6%-92%) | 90.2%  (84.9%-95.7%) | 79.1%  (70.7%-88.4%) | 95.5%  (89.5%-100%) | 91%  (82.7%-100%) |  |
|  | 5-year | 84.7%  （79.8%-89.8%) | 86.1%  （78.9%-94%) | 73.1%  （62.3%-85.7%) | 95.5%  （89.5%-100%) | 91%  （82.7%-100%) |  |
| AT endpoint | 1-year | 97.8%  （96.3%-99.3%) | 96.2%  （93%-99.5%) | 97.1%  （93.9%-100%) | 100%  （100%-100%) | 100%  （100%-100%) | 0.039* |
|  | 3-year | 95.4%  (93%-97.8%) | 95.3%  (91.6%-99%) | 91.4%  (85.4%-97.9%) | 97.9%  (94%-100%) | 100%  (100%-100%) |  |
|  | 5-year | 94.1%  （90.8%-97.6%) | 95.3%  （91.6%-99%) | 86.9%  （76.9%-98.1%) | 97.9%  （94%-100%) | 100%  （100%-100%) |  |
| DVT endpoint | 1-year | 97.8%  （96.3%-99.3%) | 97.8%  （95.3%-100%) | 92.7%  （87.9%-97.7%) | 100%  （100%-100%) | 94%  （87.6%-100%) | 0.100 |
|  | 3-year | 95.4%  (93%-97.8%) | 94.7%  (90.5%-99%) | 87.3%  (80.5%-94.6%) | 95.5%  (89.5%-100%) | 91%  (82.7%-100%) |  |
|  | 5-year | 94.1%  （90.8%-97.6%) | 90.4%  （83.6%-97.8%) | 84.9%  （77.1%-93.6%) | 95.5%  （89.5%-100%) | 91%  （82.7%-100%) |  |

**P*＜0.05: log–rank test.

*AT*, arterial thrombosis; *DVT*, deep venous thrombosis; *CI*, confidence interval.
